# Conception rate, uterine infection and embryo quality after artificial insemination and natural breeding with a stallion carrier of *Pseudomonas aeruginosa*: a case report

**DOI:** 10.1186/1751-0147-54-20

**Published:** 2012-03-29

**Authors:** Guimarães Tiago, Carvalheira Júlio, Rocha António

**Affiliations:** 1 ICBAS - Abel Salazar Biomedical Institute, ICBAS, University of Porto, Campus Agrário de Vairão, Rua Padre Armando Quintas 7, Vairão 4485-661, Portugal; 2 CECA/ICETA - Animal Sciences Centre, ICBAS, University of Porto, Campus Agrário de Vairão, Rua Padre Armando Quintas 7, Vairão 4485-661, Portugal; 3 CIBIO/ICETA - Research Centre for Biodiversity and Genetic Resources, ICBAS, University of Porto, Campus Agrário de Vairão, Rua Padre Armando Quintas 7, Vairão 4485-661, Portugal

**Keywords:** Stallion, *Pseudomonas aeruginosa*, Venereal diseases, Artificial insemination, Embryo quality

## Abstract

**Background:**

*Pseudomonas aeruginosa *may cause venereal disease and infertility in horses. A *Pseudomonas aeruginosa *- carrier stallion, often unresponsive to artificial vagina collection, was used to naturally breed mares. Semen collected from the same stallion was also used to perform artificial inseminations. Pregnancy rates, embryo quality and incidence of uterine infection were compared between inseminated or naturally-bred mares.

**Methods:**

*P. aeruginosa *was isolated from swabbing of the penis, prepuce and distal urethra of the stallion. Before being bred or inseminated, clitoral/vestibular samples were collected from all mares, and cultured for isolation of *P. aeruginosa*. At the first observed estrus, endometrial swabs were also collected. All mares subjected to natural mating (NS) were re-evaluated for *P.aeruginosa *by culture of clitoral and endometrial swabs. Artificial inseminations (AI) were performed either with fresh-extended semen (11 AI/7 mares) or frozen semen (10 AI/7 mares). The stallion was also used to breed 3 mares (4 services). For embryo collection, 2 mares were inseminated with fresh-extended semen (1 AI/mare), and 2 additional mares were inseminated with frozen semen (2 AI/mare). Two mares were naturally-bred with a total of 9 services, for embryo collection. All mares were examined after AI or natural service (NS), for uterine pathologies. Embryo recoveries were attempted passing a catheter with inflatable cuff connected to a sterile flexible 2-way flushing catheter, through the cervix. Flushed media was recovered into an Em-Con filter, and embryos searched using a stereoscope. Embryos were graded from 1 (excellent) to 4 (degenerated/dead).

**Results:**

Pregnancy rates obtained after NS was 50% per cycle. However, more than half of the NS resulted in uterine disease, while uterine pathology was seen only in 22% of the time following AI. Half of the mares bred by NS got positive to *P. aeruginosa*. Percentage of embryo recovery rates was identical after AI or NS (66.7%). The 4 embryos recovered after AI were classified as Grade 1, while after NS only 2 out of the 6 recovered embryos were Grade 1.

**Conclusion:**

a) there was no evidence of reduced fertilization after AI or NS, b) a numerically higher incidence of uterine disease was noticed after NS, c) venereal transmission of *P. aeruginosa *after NS was confirmed, d) a lower percentage of G1 embryos may be obtained after NS. Overall, the data supports the indication for *P. aeruginosa*-carrier stallions to be bred by AI rather than by NS, and raises the possibility that *P. aeruginosa *may affect embryo quality.

## Background

Horses may harbor *Pseudomonas aeruginosa *in the genitalia in percentages that ranged from 4% to 10% in mares and as high as 36% in stallions [1,2]. *P. aeruginosa *can cause venereal disease and infertility in the equine [3,4] and it is generally accepted that stallions contaminated with this bacteria need to be considered potential carriers of a venereal-transmitted disease [5], in particular when used to breed older mares [2]. *Pseudomonas aeruginosa *is frequently multi-resistant to antibiotics and is the most potent biofilm - producing pathogen of the equine reproductive tract [6]. Washing of the stallion penis with disinfectants may lead to unwanted colonization by yeasts and pathogenic bacteria. Therefore, artificial insemination (AI) associated with minimum contamination techniques is considered to have a distinct advantage over natural service to control the transmission of these bacteria [3]. However, the effect of those techniques on the control of the disease and even the rate of transmission by infected stallions after natural service (NS) is poorly documented and the effect of NS by *P. aeruginosa*-carrier stallions in embryo quality have not been reported. In the present work we decribe the conception rates obtained after NS with a stallion carrier of *P. aeruginosa*, the conception rates after AI with fresh extended and frozen/thawed semen of the same stallion, the incidence of uterine disease with either method of breeding, as well as embryo recovery rates and embryo quality after AI and NS.

## Materials and methods

*Pseudomonas aeruginosa *was repeatedly (6 times) isolated from swabbing of the penis, prepuce and distal urethra in a Holstein stallion stationed at the Centre of Animal Reproduction of Vairão, ICBAS, University of Porto. Undiluted samples from eight different ejaculates collected with artificial vagina (Missouri model) after cleaning of the stallion's penis with warm water were negative for *P. aeruginosa*. Also negative were 3 samples of semen from the same ejaculates, extended with antibiotic-free extender (E-Z Mixin^®^, Animal Reproduction Systems, Chino, CA, USA), and 6 samples of semen frozen with Botu-crio^®^, which contains amikacin. However, at the end of the breeding season one more sample collected from the stallion's genitalia resulted positive to *P. aeruginosa*. Antibiotic susceptibility tested by the disk diffusion method showed that the bacteria were multi-resistant to 21 of the 25 antibiotics used. No inflammatory cells were detected in the ejaculates and ultrasound examination (Aloka *Prosound 2 *with 5 and 7.5-10 MHz probes, Aloka Holding Europe, Steinhausertrasse, Switzerland) of the bulbourethral glands, prostate, seminal vesicles and ampullae did not evidence any sign of pathology. Successful semen collection with artificial vagina (AV) was intermittent, and the stallion's owner expressed his desire to have his mares bred by NS when a successful collection with AV could not be obtained, despite being aware of the potential risk of P. aeruginosa transmission. Ejaculates (n = 8) had a mean (± SD) volume of 182.5 ± 67.1 mL (gel-free), with a concentration of 103.4 ± 33.0 sperm cells/mL, with a subjective (optical microscopy) progressive motility of 53.1% ± 5.3%. Three ejaculates evaluated by a computer assisted sperm analysis system (ISAS^®^, Proiser) had a progressive motility of 34.6% ± 16.8%, a total motility of 74.5% ± 14.1%, 21.1% of sperm cells with rapid movements (> 25 μm/s), a linear velocity (VSL) of 25.8 ± 8.8 μm/s, a curvilinear velocity (VCL) of 66.7 ± 23.4 μm/s, and a mean velocity (VAP) of 40.5 ± 11.6 μm/s.

All mares had an intensive sports background, were 12 to 18 years old and had not been bred for at least several years.

Before being bred or inseminated with the stallion, clitoral/vestibular samples were collected from all mares at the time of admission in the center, and cultured for isolation of *P. aeruginosa*. At the first observed estrus, endometrial swabs were also collected. For clitoral/vestibular cultures, a sterile swab was utilized and the ventral aspect of vestibule, as well as the central clitoral fossa was swabbed. Endometrial swab was performed in mid-to-late estrus, utilizing a guarded swab (Ref. 17214-2950, Minitüb Ibérica, Barcelona, Spain), passed through the cervix into the uterine lumen, protected in a sterile gloved hand. The swab was then retracted into its sterile sheath. Clitoral/vestibular and endometrial swabs were placed immediately into a sterile centrifuge tube with transport medium and sent refrigerated to the university's microbiology lab, in less than two hours. All mares subjected to natural mating with this stallion were re-evaluated for *P.aeruginosa *by culture of clitoral and endometrial swabs.

Mares were teased every other day using a stallion. After signs of behavioral estrus, the mares' uterus and ovaries where examined daily by ultrasound scanning (Aloka *Prosound 2*, 5 and 7.5-10 MHz probes) and 1500 UI of human chorionic gonadotropin (hCG - Chorulon^®^, Intervet, Portugal) was administered (iv) after detection of a follicle with a diameter ≥ 35 mm. Twenty four hours later semen was collected after cleaning of the penis with warm water and diluted at a concentration of 50 × 10^6 ^sperm cells/mL in antibiotic-free extender (E-Z Mixin^®^, Animal Reproduction Systems, Chino, CA, USA), and AI was performed with approximately 450 to 600 × 10^6 ^sperm cells with progressive motility. Artificial insemination with frozen semen was performed within 6 hours post ovulation with approximately 300 × 10^6 ^sperm cells with progressive motility. The stallion was also used to breed mares by NS. For that, the same management used for AI including the cleaning of the stallion's penis was used, with the AI replaced by NS. No filling of the uterus with extender immediately prior to NS as described originally for the minimum contamination technique [7] was done, to allow for the confirmation of ejaculated semen in the uterus by ultrasound scanning. Ultrasound examinations were repeated daily after AI or natural service to confirm ovulation and to assess uterine clearance until day 4 post-ovulation.

When uterine fluid was present 24 hrs post AI (delayed uterine clearance/endometritis) mares were treated with 30 IU of oxytocin i.v. (Placentol^®^, CEVA, Portugal) 2 to 3 times daily and the uterus was flushed with saline. One mg/day of Cetiofur (Excenel^®^, Pfizer Manufacturing, Puurs, Belgium) was infused in the uterus up till day 4 post ovulation when purulent uterine exudates were recovered after uterine flushing, or when a neutrophile-positive uterine swabbing was obtained 24-hrs after AI, and in all mares bred by NS.

Eleven inseminations were performed with fresh-extended semen in 7 mares. Ten inseminations were performed with frozen semen in 7 mares. Three mares were hand mated by NS to get impregnated, two of which in a single estrous cycle and other in two estrous periods. One of these mares had turned positive to *P. aeruginosa *after previously being bred by NS for embryo collection. Ultrasound examination confirmed pregnancies 12 to 16 days post ovulation.

For embryo collection, 2 mares were inseminated with fresh-extended semen (1 AI/mare), and 2 additional mares were also inseminated with frozen semen (2 AI/mare), in two consecutive cycles. Two mares were allocated to be bred by NS for embryo collection. A total of 9 natural services were performed in these 2 mares. Embryo recoveries were attempted passing a catheter with inflatable cuff through the cervix, connected to a sterile flexible 2-way flushing catheter, after careful washing of the vulva. Once in the body of the uterus, the cuff was inflated with 50 to 70 mL of air and the catheter pulled backwards onto the internal os of the cervix to seal off the uterus. Between 2 and 3 Lts of warmed Ringer lactate (Laboratorio sorologico, Amadora, Portugal) for embryo collections were infused into the uterus. The media was recovered by gravity flow into an Em-Con filter, after which the cuff was deflated to allow the withdrawal of the catheter. The medium in the filter was than transferred to a Petri dish and embryos searched using a stereoscope. The embryos were graded from 1 (excellent) to 4 (degenerated/dead) as reviewed by McCue *et al. *[8].

All statistical procedures for analysis of the data were computed using SAS software [9]. Fisher's exact test was dimmed the appropriate statistic to assess the univariate associations between the outcome of interest (positive vs negative to AI with fresh and frozen semen and NS) with pregnancy per cycle, uterine pathology and frequencies of Grade 1 embryos. Differences were considered significant when P < 0.05.

Authorization was granted, from the owner of the animals, to use the data for a scientific publication.

## Results

### Pregnancy rates; metritis/delayed uterine clearance

Pregnancy rates per cycle were not significantly different (P > 0.3). Data on pregnancy rates obtained after AI and NS are detailed in Table 1. All 7 mares inseminated with fresh-extended semen became pregnant. Only one of these mares required post-AI treatment for delayed uterine clearance. No delayed uterine clearance was detected in the 2 mares inseminated with fresh-extended semen for embryo collection, and one embryo was collected.

**Table 1:** Pregnancy rates after AI with fresh extended and frozen thawed semen, and after natural service

	N° of Mares	N° of Services	Pregnancy per cycle (%)	Total pregnant mares (%)	IA/Pregnancy*
AI fresh extended	7	11	7/11 (64)	7/7 (100)	1.57

AI frozen/thawed	7	10	4/10 (40)	4/7 (57)	2.5

Natural Service	3	4	2/4 (50)	2/3 (67)	2.0

* Includes mares that did not get pregnant

Four of the 7 mares inseminated with frozen semen got pregnant (57%). Embryos were recovered in 3 out of 4 flushings (75% recovery rate).

Two of the 3 mares bred by NS became pregnant. *P. aeruginosa *was isolated from one pregnant mare (clitoral swab) and from one of the mares that did not get pregnant (clitoral and uterine swab). Embryos were collected in 6 out of 9 flushings (66% recovery rate).

Differences in uterine pathology between mares inseminated or subjected to NS approached significance (P = 0.07). In total, 6 cases (22%) of delayed uterine clearance/endometritis were noted after the 27 AIs performed with fresh or frozen/thawed semen, either for pregnancy or for embryo collection, while 7 cases (54%) of endometritis were diagnosed after the 13 natural services performed by the stallion for the establishment of pregnancies or for embryo collection.

### Embryo quality

The difference in frequency of Grade 1 embryos produced by AI or NS approached significance (P < 0.08). The embryo recovered after AI with fresh semen was classified as a day 8, Grade 1 blastocyst. Two day 8 Grade 1 blastocysts and a day 10 Grade 1 expanded blastocyst were obtained after insemination with frozen semen. In relation to embryo quality after natural breeding, the results were as follow: one Grade 4 morula after a day 8 flushing, a Grade 2 morula and a grade 1 blastocyst after a day 8 flushing of a mare with two ovulations within a 3 day interval, a Grade 2 and a Grade 4 blastocysts in two nine-day flushings, respectively, and a Grade 1 expanded blastocyst after a day 10 flushing.

Table 2 summarizes the results obtained in relation to embryo recovery rates and embryo quality after NS or AI.

**Table 2:** Embryo recovery attempts, embryo recovery rates and embryo quality after Artificial Insemination (fresh extended plus frozen/thawed semen) or Natural Service

	N° of Mares	N° of Services	Embryos/Flushing (%)	Grade 1 Embryos/Total Embryos (%)	Grade 2 Embryos/Total Embryos (%)	Grade 3-4 Embryos/Total Embryos (%)
AI	4	6	4/6 (66.7)	4/4 (100)	---	---

Natural Service	2	9	6/9 (66.7)	2/6 (33.3)	2/6 (33.3)	2/6 (33.3)

## Discussion

The results of this study were based on a limited number of observations and thus no solid inferences can be made about the fertilizing ability of inseminated semen *vs*. NS for this stallion. Notwithstanding that, conception rates and embryo recovery rates after AI with fresh and frozen semen were in the normal range obtained in our centre. Not all ejaculates were tested for the presence of *P. aeruginosa*, but the fact that the bacteria was not isolated from 8 fresh, undiluted ejaculates raises the possibility that it was not present in the semen or had a very low concentration. As no isolations were obtained in undiluted semen, none should be expected in extended semen even if without antibiotics (E-Z Mixin^®^) or even less when containing antimicrobials (Botu-crio^®^). The somewhat lower conception rate achieved with frozen semen may be attributed, at least in part [10], to degenerative conditions of the endometrium of two older mares (18 years-old) which had a grade III Kenny's endometrial classification. Pregnancy rates as well as embryo recovery rates obtained after NS with the infected stallion were acceptable despite having more than half of the NS resulting in uterine disease compared to only in 22% following insemination. These positive results may have been due, at least in part, to the intensive uterine treatments performed right after every natural service, up till day 4 post ovulation. Confirmation of venereal transmission of *P. aeruginosa *and was obtained with 2 out of the 4 mares bred by natural service getting positive to *P. aeruginosa *and thus acquiring the potential to transmit the bacteria.

The majority of recovered equine embryos were of good to excellent quality [8]. In the present case all embryos collected after AI were of excellent quality. However, and despite identical embryo recovery rates after AI or NS, embryo quality was decreased after the latter, compared to AI. In cattle, the use of *P. aeruginosa *- contaminated semen did not result in decreased embryo quality [11]. However, in cattle as opposed to in horses, *P. aeruginosa *it is not considered to be a potentially venereal transmitted disease. Taken together the data suggest that NS with a *P. aeruginosa*-carrier stallion resulted in acceptable fertilization rates, but despite aggressive post-insemination treatments originated a higher percentage of lower quality embryos.

## Conclusions

With this carrier stallion, there was no evidence of low fertilization rates after AI or NS, but uterine disease was noted more often after NS than after AI. Venereal transmission of *P. aeruginosa *after NS did occur, and was associated with lower embryo quality after NS compared to AI. Overall, the data supports the indication for *P. aeruginosa *- carrier stallions to be bred by AI rather than by NS, both when used to impregnate mares or to breed embryo donors.

## Competing interests

The authors declare that they have no competing interests.

## Authors' contributions

AR and TG executed the field work and collected the data, TG wrote the first draft of the manuscript, AR and JC produced the final version of the manuscript. All authors read and approved the final manuscript.
